# Comparative Clustering of Plantar Pressure Distributions in Diabetics with Polyneuropathy May Be Applied to Reveal Inappropriate Biomechanical Stress

**DOI:** 10.1371/journal.pone.0161326

**Published:** 2016-08-16

**Authors:** Uli Niemann, Myra Spiliopoulou, Thorsten Szczepanski, Fred Samland, Jens Grützner, Dominik Senk, Antao Ming, Juliane Kellersmann, Jan Malanowski, Silke Klose, Peter R. Mertens

**Affiliations:** 1 Faculty of Computer Science, Otto-von-Guericke University, Magdeburg, Germany; 2 ifak system GmbH, Magdeburg, Germany; 3 mediXmind GmbH, Magdeburg, Germany; 4 Clinic for Nephrology, Hypertension, Diabetes and Endocrinology, Otto-von-Guericke University, Magdeburg, Germany; University of Louisville, UNITED STATES

## Abstract

In diabetic patients, excessive peak plantar pressure has been identified as major risk factor for ulceration. Analyzing plantar pressure distributions potentially improves the identification of patients with a high risk for foot ulceration development. The goal of this study was to classify regional plantar pressure distributions. By means of a sensor-equipped insole, pressure recordings of healthy controls (n = 18) and diabetics with severe polyneuropathy (n = 25) were captured across eight foot regions. The study involved a controlled experimental protocol with multiple sessions, where a session contained several cycles of pressure exposure. Clustering was used to identify subgroups of study participants that are characterized by similar pressure distributions. For both analyzed groups, the number of clusters to best describe the pressure profiles was four. When both groups were combined, analysis again led to four distinct clusters. While three clusters did not separate between healthy and diabetic volunteers the fourth cluster was only represented by diabetics. Here the pressure distribution pattern is characterized by a focal point of pressure application on the forefoot and low pressure on the lateral region. Our data suggest that pressure clustering is a feasible means to identify inappropriate biomechanical plantar stress.

## Introduction

The diabetic foot syndrome has a substantial impact on a patient’s quality of life [1]. Next to being associated with an increased mortality [1, 2], it increases the risk of foot ulceration [3], whereby the rate of foot amputations among diabetics has been estimated to be 17–40 times higher than for non-diabetics [4].

Elevated plantar pressure has been early on identified as a major risk factor for ulceration among diabetic patients [5–7]. Customized footwear and orthopedic footwear are since then developed as means to prevent ulceration and for personalized therapy [8–10].

A pressure threshold with high sensitivity and specificity towards ulcer development has not yet been determined [11]. Such a threshold would be computed by aggregating and smoothing plantar pressure over the whole foot. More recently, researchers also investigate how pressure is applied on each foot region. Bennetts et al. point out that there are differences among foot types and foot biomechanics, leading to differences in pressure distribution among regions of the same foot [12]. Deschamps et al. bring forward “the stratification of patients based on their plantar pressure pattern homogeneity (biomechanical approach)” as an approach that may avoid the pitfall of smoothing away variations within a pathophysiological group [13].

Clustering is the method of choice for deriving groups of patients that exhibit similarities in pressure distribution, mostly concentrating on *peak plantar pressure* (in most citations this term has been been defined as the maximum observed pressure recorded for a single measurement, e.g. the maximum pressure at a sensor during a step) [12–14], discussed hereafter. De Cock et al. [14] studied the peak pressure recorded on different foot regions during jogging, and identified four pressure patterns that differ on the focal point of pressure. For example, the “M2 pattern” is the cluster where the maximum of mean total regional impulse among plantar regions is at the second metatarsal region. Bennetts et al. [12] considered seven plantar regions and built groups of patients that exhibit similar peak plantar pressure distributions among these regions. Deschamps et al. [13] concentrated on peak plantar pressure distribution of the forefoot only, studying both patients and controls. They built clusters of patients with similar pressure distributions of the forefoot, and identified one cluster that only consists of diabetic patients [13].

In the present study, pressure distributions among diabetics with severe polyneuropathy, among healthy controls and differences between patients and controls in a controlled experiment were investigated. Similarly to [12], regions from all areas of the foot were considered, including the heel. Since the experiment only involves standing and being seated, and no walking at all, peak plantar pressure was not considered, as the aforementioned studies do. Rather the pressure observed for each participant during the experiment was normalized, taking the recorded extrema (minimum and maximum) into account.

## Methods

### Subjects

The total diabetic population chosen for this study consisted of 25 participants (=DiabGr, 6 females, 19 males, age 64.8 ± 9.8 years). In addition to the diabetic group, 18 non-diabetic volunteers (=ContrGr, 10 females, 8 males, age 62.9 ± 7.6 years) were considered.

All participants of the study provided informed written consent. Inclusion criteria for healthy volunteers were the absence of macroangiopathy (determined by ABI measurements), skin defects of lower extremities, sensomotoric neuropathy (determined by Rydel/Seiffer vibration, tip-therm and 10-g Semmes-Weinstein monofilament examination, achilles and patellar tension reflexes, overall strength of extensors and flexors), exclusion of diabetes in past medical history, lack of amputations of limbs/deformations of the spine, negative history of foot ulcerations, negative past medical history for heart failure, myocardial infarctions. For diabetics with proven neuropathy inclusion criteria were defined as follows: Diagnosis of type 1 or 2 diabetes by specialized diabetes health care provider, detection of peripheral sensoric neuropathy with impaired proprioception detected by graduated Rydel/Seiffer timed vibration perception performed with a 128 Hz tuning fork and a threshold of ≤ 2/8, absence of thermal distinction assessed by tip-therm testing and negative 10-g Semmes-Weinstein monofilament perception. Patellar and achilles tension reflexes were tested positive in all included diabetics, however to different degrees, as was overall muscle strength for plantar pressure and flexor/extensor muscle strengths. No major movement impairment was detected in participants. Neuropathic ulcerations (current or in status nascendi) and other skin defects as well as major macroangiopathies (Fontaine stage II and higher) were excluded in all diabetics. Further exclusion criteria encompassed paralysis of lower extremities, heart failure stages III and IV (NYHA), amputations of limbs/deformations and myocardial infarction within the preceding 12 weeks.

The study protocol was approved by the Ethics Commission of the Otto-von-Guericke University Magdeburg at the Medical Faculty and the University Clinic. According to EUDAMED CIV-13-06-011441, the study protocol was exempted from approval for medical devices with low safety risk.

**Fig 1:**
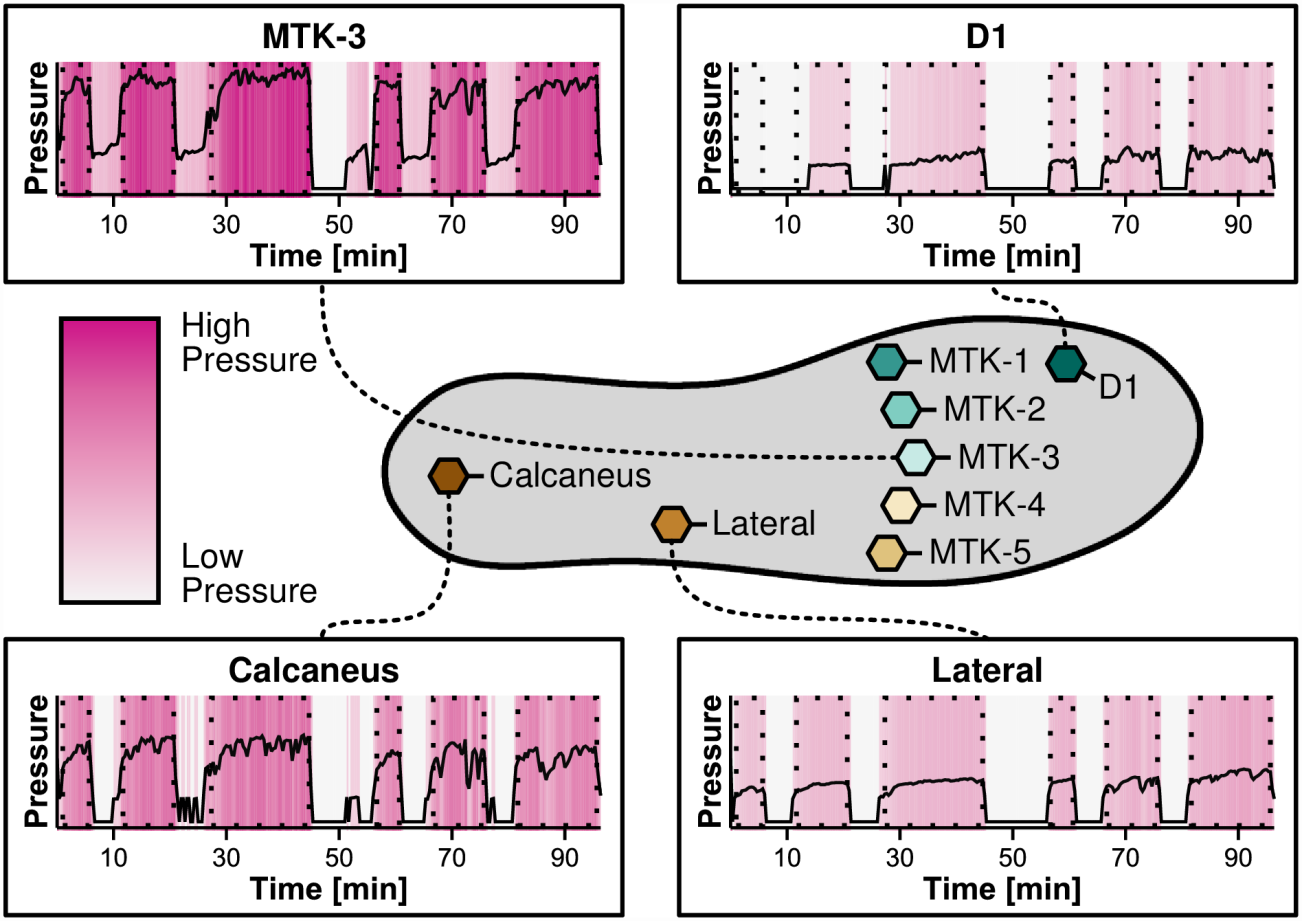
Insole sensor locations and pressure time curve examples. Sensor locations in relation to the insole (center) and four pressure time curves of representative foot regions derived from the study for an example patient. Time intervals where the patient was asked to stand and apply pressure are highlighted by dotted rectangles. These lasted over 5, 10 and 20 minutes, respectively. D1: Digitus-1; MTK-1 to 5: Metatarsal Bone 1 to 5.

A dedicated insole [15] was used to record plantar pressure loads: a flat, evenly deep indentation (*profile*) is worked into the surface of the insole and incorporates a measurement system to which multiple pressure and temperature sensors are connected. The sensor placement is based on the knowledge of critical regions prone to foot deformity [16]. In particular, the insole consists of eight distinct temperature sensors and eight distinct pressure sensors placed on the upper surface of the insole to allow close contact to the participant’s foot; a schematic representation of the insole sketching the sensor locations is depicted in Fig 1 (center).

### Instrumentation and Protocol

The participants were asked to wear the insole for 100 minutes while performing a “session” composed of two postures:

- Seated: the experiment participants were seated, resting their feet (on the ground).
- Standing: the experiment participants were standing upright and applying pressure on both feet.

The procedure was as follows: seated 5 minutes, standing 5 minutes, seated 5 minutes, standing 10 minutes, seated 5 minutes, standing 20 minutes. For all 43 sessions, sensor recordings of both feet were available which were used independent, reaching a total of 86 session datasets, which were used in the analysis. Raw sensor values were recorded at a time interval of 20 seconds (for 30 of the 86 datasets) or 3 seconds (for the remaining 56 sessions), respectively (see S1 Datasets).

### Data Analysis I—Sensor Data Preparation

The pressure recording for one foot of an example participant is depicted in Fig 1: the pressure of the session constitutes one time-series per sensor. Within each time-series, the phases in which the patient was standing are indicated by dotted rectangles. During these phases, the example patient applied more pressure to the central regions MTK-3 and Calcaneus than to Digitus-1 and Lateral indicating that this patient distributed pressure in a balanced way with focal point of pressure to the central regions.

Some of the sensor recordings were identified as noisy or erroneous. To reduce the effects of such *extreme* recordings, these values were replaced with the median of all recordings for the sensor of the respective session. A recording *r* was defined as extreme if *r* < *Q*_1_ − 1.5 × *IQR* or *r* > *Q*_3_ + 1.5 × *IQR* where *Q*_*i*_ is the *i*^*th*^ quartile and *IQR* is the interquartile range, *IQR* = *Q*_3_ − *Q*_1_. Subsequently, the time curves were smoothened using *locally weighted scatterplot smoothing* (LOWESS) [17] with a smoother span of 5%.

The pressure recorded during the “Seated” phases was zero: this was the case for all 86 sessions, i.e. for both feet of all participants, and for all three “Seated” phases in each session. Therefore all “Seated” phases have been excluded from the analysis of plantar pressure. For each session *i* (i.e. for each participant and foot separately), the minimum and maximum observed pressure values over all sensors were identified, *r*_*i*,*min*_ and *r*_*i*,*max*_ respectively. Then, within each session *i* and for each sensor *s* each observed pressure value *r*_*i*,*s*_ was normalized into the *relative plantar pressure* (RPP) value $r_{i,s}^{*}=\left(r_{i,s}-r_{i,min}\right)/\left(r_{i,max}-r_{i,min}\right)$, and the median *median*(*i*, *s*) for each sensor *s* over all “Standing” phases of session *i* was derived. The median was opted instead of the average, to minimize the impact of outlier values, that might be caused e.g. through unconscious movements of the participants while they were standing. Thus, a session *i* was modelled as a vector of the 8 median values of the sensors.

To build clusters of sessions where participants distributed the pressure they applied in a similar way across all regions, we defined session similarity on the basis of the Euclidean norm: the distance *d*() between two sessions *i*, *j* is

$$d\left(i,j\right)=\sqrt{\sum_{s\in S}\left(median\left(i,s\right)-median{\left(j,s\right)}^{2}\right)}$$ (1)

where *S* is the set of all 8 sensors we consider (at the regions MTK-1…MTK-5, Digitus-1, Calcaneus, Lateral, cf. Fig 1).

### Data Analysis II—Clustering

For the partitioning into groups of plantar pressure distributions, *k*-medoids clustering was applied [18]. In *k*-medoids clustering, a cluster is defined as a set of instances where one of the instances is the representative, the so called “medoid”. The algorithms proceeds as follows. First, *k* instances are randomly selected as initial medoids. Each of the remaining instances is assigned to the cluster with medoid nearest to it. Then, each cluster is updated by choosing a new medoid, the one that minimizes intra-cluster distance. The next medoid (from 2 to *k*) is the instance that minimizes the sum of distances to all other instances. All instances are then reassigned with respect to the updated medoids. The iterations continues until the cluster contents do not change from one iteration to the next. Since the random initialization phase may produce different results, an implementation of *k*-medoids was used that has a “build phase”, where it identifies an appropriate initial set of medoids [19]. The instance is chosen that minimizes the sum of distances to all other instances.

*k*-medoids clustering was run for *k* = {2, 3, …, 10}. The optimal number of clusters was determined by the Silhouette coefficient *Silh* which is defined as

$$Silh\;=\;1/n\sum_{p=1}^{n}\frac{b(p)-a(p)}{\text{max}\left\{a(p),b(p)\right\}},$$ (2)

where *a*(*p*) is the average distance between a instance *p* and all other instances of the same cluster (cluster peers), *b*(*p*) is the average distance from *p* to the instances of the nearest cluster and *n* is the total number of instances in the dataset. The value of *Silh* ranges from −1 to 1. Values near 1 are preferred since they express a high compactness of cluster peers and a large distance of instances to other clusters. When on average instances are closer to another cluster’s instances than to their cluster peers, *Si* becomes negative indicating that the cluster membership assignment is inappropriate.

Clustering was consecutively performed for the diabetic group (DiabGr, number of session datasets *n* = 50), controls (ContrGr, number of session datasets *n* = 36) and a combination of both groups where a random sample of DiabGr was randomly selected (BothGr, number of session datasets *n* = 36 + 36 = 72). Clustering and best clustering selection have been performed in R version 3.2.2.

## Results

**Fig 2:**
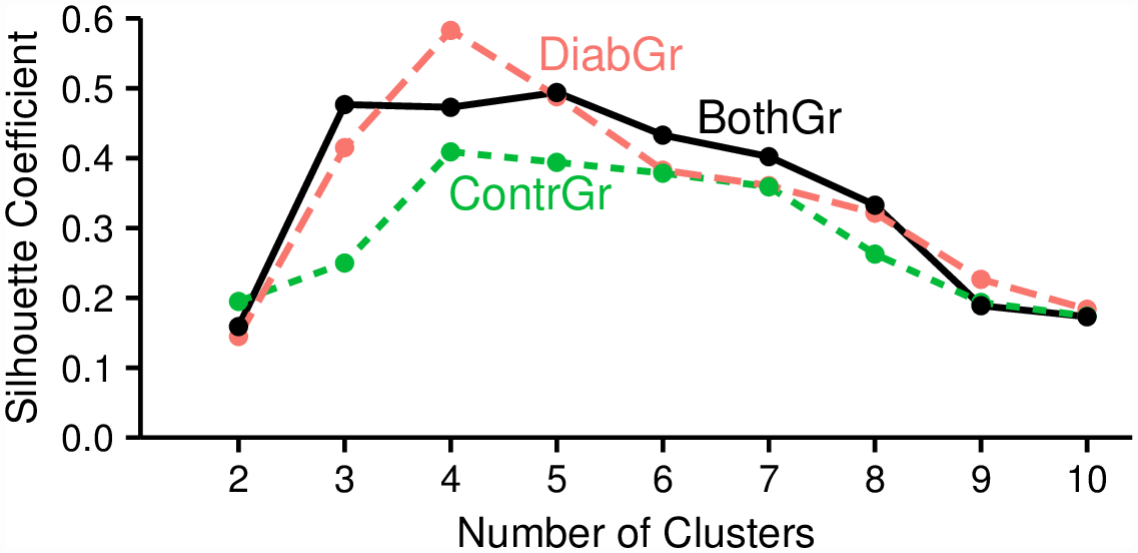
Quality Assessment of *k*-medoids clustering using the Silhouette coefficient. Silhouette coefficients for *k*-medoids clustering using the distribution of eight plantar pressure regions with the number of clusters *k* set between 2 and 10 for each group. For each group best clustering is achieved with *k* = 4 clusters.

**Fig 3:**
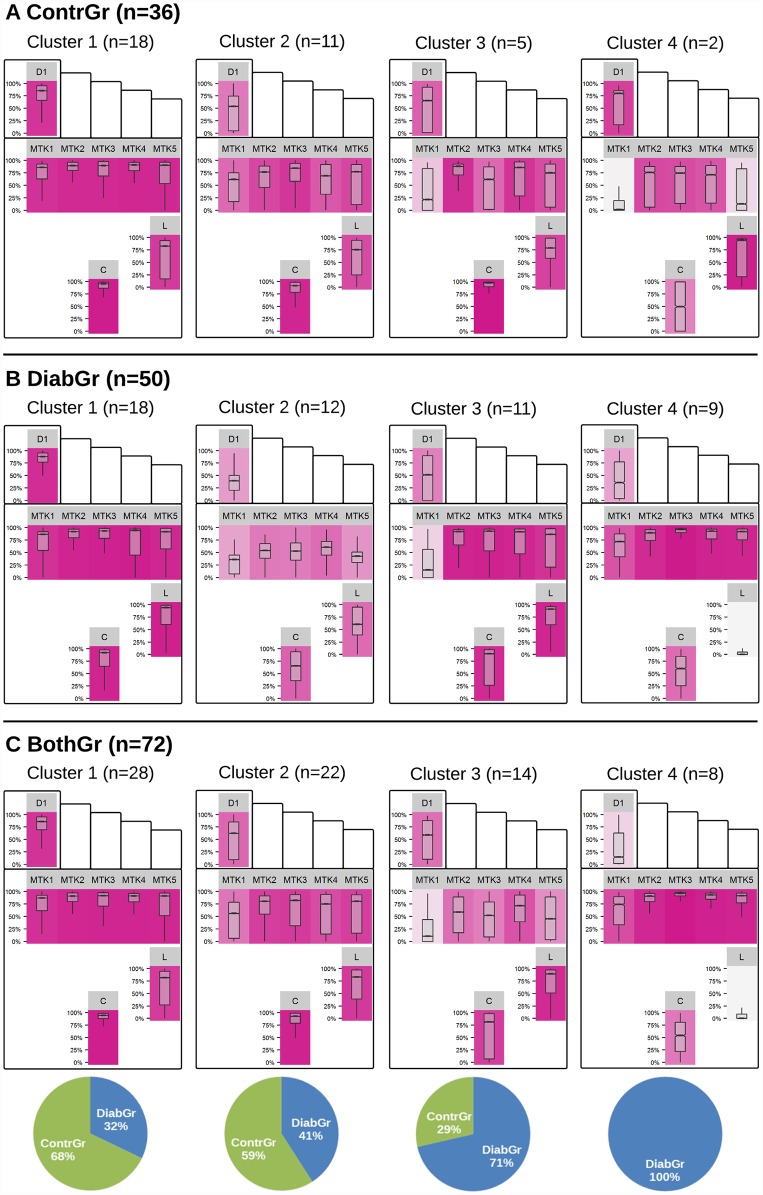
Summary of the clusters’ relative plantar pressure distribution. Relative plantar pressure distribution for each cluster and region (standing and sitting). The color of a panel’s background reflects median relative plantar pressure with a linear color gradient, from light gray (low relative plantar pressure) to violet (high relative plantar pressure). Pie charts depict the relative portion of ContrGr and DiabGr session datasets for each of BothGr’s clusters. D1=Digitus-1, L=Lateral, C=Calcaneus.

Fig 2 shows the optimal number of clusters for each group. Best silhouette coefficient values were achieved for *k* = 4 in all experiments, which means that the patients, the controls and both groups together can be best described with 4 plantar pressure profiles. For the clustering with the optimum *k* of each group, a summary of the relative plantar pressure distribution for the session datasets of each cluster is provided in Fig 3.

For ContrGr, the optimum number of clusters was four. For cluster 1 which represents 50% of the controls population, median relative plantar pressure is high throughout all regions. Clusters 2 and 3 show some variability for the pressure load on the forefoot regions. Cluster 4, characterized by low median relative plantar pressure at MTK-1 and MTK-5, describes only 2 out of 36 feet from ContrGr.

For DiabGr, four distinct pressure distributions were identified by *k*-medoids with cluster 1 being the largest that is characterized by high plantar pressure (RPP above 80%). Cluster 2 represents evenly balanced, intermediate relative plantar pressure profiles. Here, the median relative plantar pressure values for Digitus-1, MTK-1 and MTK-5 ranges between 30% and 50% of the maximum whereas median relative plantar pressure for the central forefoot regions (MTK-2, MTK-3, MTK-4), Lateral and Calcaneus are sensed between 50% and 75%. For cluster 3, median relative plantar pressure is above 80% of maximum values for all regions except for the medial regions Digitus-1 and MTK-1. The latter cluster exhibits the largest variance of all four clusters, with a high spread between first and third quartile for several regions. For cluster 4 high median relative plantar pressures are sensed on all MTK locations (relative plantar pressure > 75%), and very low pressure on Lateral (median RPP < 2%).

When we mix the two groups of participants in BothGr, some clusters of the diabetics merge with the ones of controls. The optimal number of clusters to describe the pressure distribution best remains 4, as can be seen from the values of the silhouette coefficient in Fig 2. From these four clusters, three (clusters 1–3) contain both patients and controls, but cluster 4 summarizes pressure distribution patterns only found in diabetics with severe polyneuropathy.

Table 1 summarizes the data for diabetics and controls, as they are determined within the four clusters of BothGr. For clusters 1–3, the means of weight and BMI for diabetics is higher than that of controls.

**Table 1:** Cluster description and composition, separated by DiabGr and ContrGr.

	Cluster 1		Cluster 2		Cluster 3		Cluster 4	
	Diabetics	Controls	Diabetics	Controls	Diabetics	Controls	Diabetics	Controls
Number of feet	9	19	9	13	10	4	8	0
Sex [f/m]	2/7	9/10	4/5	8/5	4/6	3/1	1/7	-
Age [years]	60.7 ± 8.8	64.3 ± 6.8	67.9 ± 4.7	59.8 ± 8.5	67.7 ± 7.6	66.5 ± 6.2	63.3 ± 10.0	-
Height [cm]	177.9 ± 4.5	171.9 ± 8.4	172.9 ± 5.2	169.2 ± 11.5	172.9 ± 6.4	165.5 ± 11.2	177.8 ± 5.9	-
Weight [kg]	97.1 ± 21.6	79.2 ± 13.0	85.1 ± 14.7	75.5 ± 10.7	92.3 ± 14.8	68.5 ± 17.6	99.9 ± 17.5	-
BMI	30.6 ± 6.3	26.6 ± 2.9	28.4 ± 4.2	26.5 ± 3.5	30.9 ± 4.6	24.9 ± 5.5	31.4 ± 4.0	-

There were no significant inter-cluster differences except for clusters 2 and 4 (height, weight and BMI) and clusters 3 and 4 (height); *α* = 0.05.

Nevertheless differences in weight and BMI alone may not explain the differences seen between the clusters. Statistics revealed that the effects of these variables were not relevant for assignment to most clusters (Table 1). Furthermore the mean weight for diabetics was higher than controls in each cluster.

**Fig 4:**
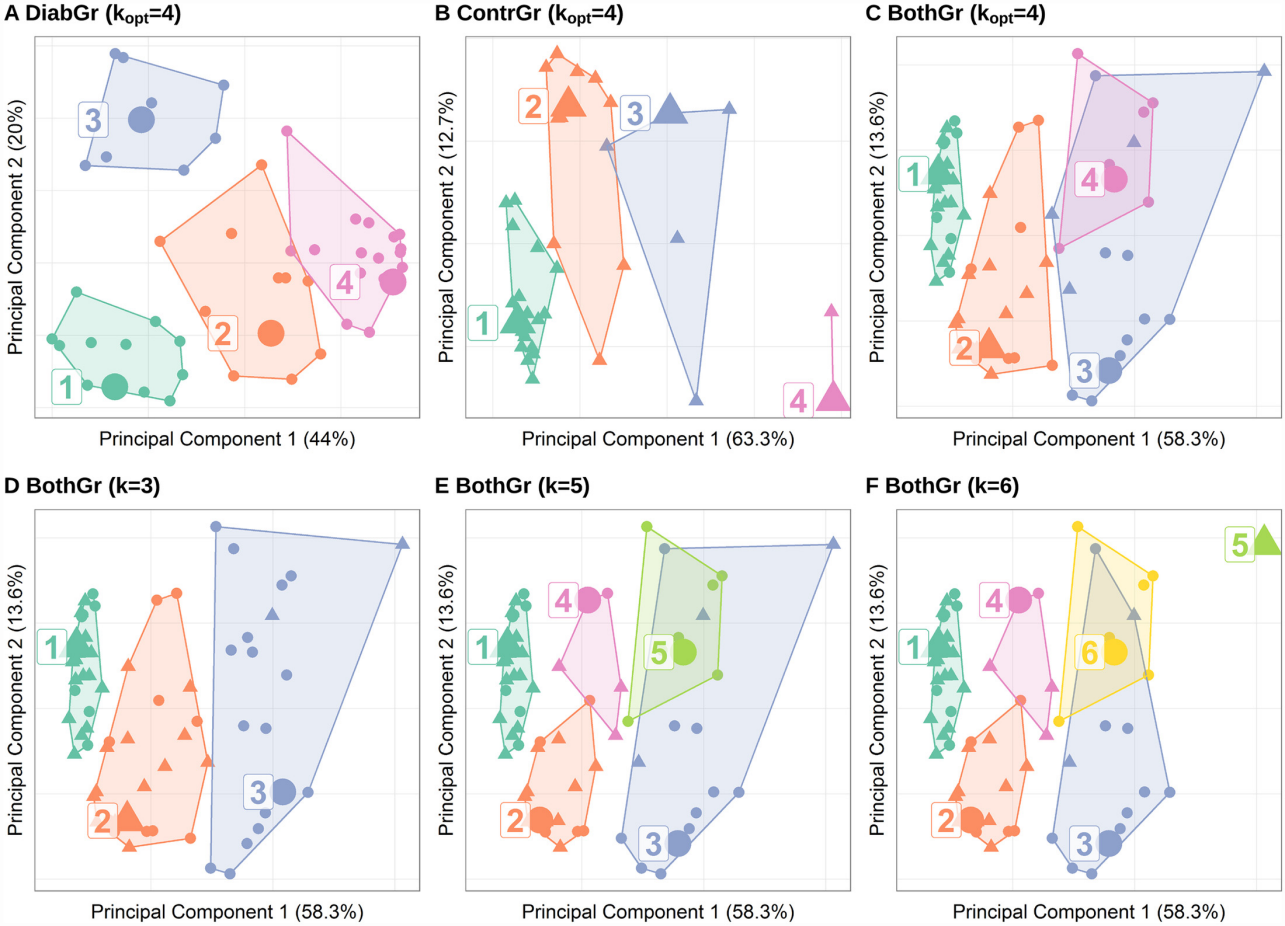
Visualization of the clusters projected in 2-D space using principal component analysis. A–C show the partitioning of *k*-medoids with *k* set to the optimal value according to Fig 2; D–F depict alternative partitionings with a different, non-optimal number of clusters on BothGr. The clusters are projected on the first two principal components of the eight-dimensional feature space. Percentages in axis titles reflect the explained variance of the principal component. Points/ triangles depict sessions from DiabGr and ContrGr, respectively. Larger symbols represent cluster medoids.

The scatterplots in Fig 4A–4C visualize the partitioning of the best clustering run for each of the three groups, projected on the first two components leveraged by application of principal component analysis (PCA) to the eight-dimensional feature space. Note that the transformation of the original variables is different for DiabGr, ContrGr and BothGr because PCA was applied to each group separately. While clustering of the DiabGr group and ContrGr yields a partitioning with separated clusters respectively, the clustering on BothGr shows some overlapping, especially between cluster 3 with cluster 4 (cf. Fig 4). Cluster 4 contains 8 sessions from DiabGr exclusively; in cluster 3 a large portion of session are of DiabGr (10 out of 14 ≈ 71%). Cluster 1 features the majority of sessions from ContrGr (19 out of 28 ≈ 68%).

## Discussion

Four pressure clusters were identified for controls and diabetics each. These clusters reflect the ways the groups distribute pressure among the regions of their feet. By combining the data it became apparent that a clear separation of diabetics from controls is only feasible in a subgroup (see Fig 3C). The ultimate goal of such profiles is to reduce the risk of ulceration: while a clinical examination is still inevitable, an analysis of pressure distribution, as proposed here, can serve as basis for preventive strategies [7, 9, 13, 16, 20–22], including the preparation of foot wear that is personalized to the patients’ needs [12].

Leveraging the silhouette coefficient as means to assess clustering quality, the optimal clustering produced by *k*-medoids was at *k* = 4 for both the control group and the diabetic group. This number is consistent with a previous study [13] (for the diabetic group) and [14]. Further, some of the results of [12] for *k* = 4 could be reproduced. For example, in [13], the pressure pattern of cluster 4 with higher pressure on the lateral forefoot regions for the diabetic group that was not present in the controls was also identified in this study (cluster 4).

The juxtaposition of clusters for ContrGr and DiabGr revealed high overlap in plantar pressure distribution. For example, cluster 1 and cluster 3 of ContrGr and DiabGr are characterized by nearly identical pressure load patterns, respectively. To investigate whether subgroup-specific pressure distribution could be identified, cluster analysis was conducted on the combination of both groups. To ensure a balanced distribution between both groups, a random sample from DiabGr was selected. The observed pressure patterns in Fig 3C support the assumption of shared pressure patterns: cluster 1, 2 and 3 comprise pressure distributions of both groups. However, cluster 4 was unique for DiabGr. This pattern is characterized by the lowest median relative plantar pressure on Lateral and Calcaneus.

Some of the observed differences in comparison with related studies are due to the disparity in study participants, population sizes, experiment protocols and measuring devices [12–14]. For example, neither the medial M1 pattern nor the M2 pattern of [14] where the focal point is on either MTK-1 or MTK-2 could be replicated in this study. Further, some of the results were not observed in past studies, e.g. the disparity between Lateral and other regions in cluster 4 (DiabGr).

Juxtaposing results for DiabGr and ContrGr, besides similar plantar pressure distributions some subgroup-specific idiosyncrasies were identified. A good indicator is the distribution of study datasets from DiabGr and ContrGr for the clusters computed for BothGr. A subgroup of high relative plantar pressure distribution over all plantar regions was found both in ContrGr and DiabGr (cluster 1, respectively). This distribution is represented by cluster 1 of BothGr which makes up for the largest of the five clusters (n = 28; ≈ 39%) and cluster 2 (n = 22; ≈ 31%). Similar to cluster 1 and 2 but with less pressure on the medial and lateral regions is cluster 3. The majority of pressure distributions of ContrGr are present in cluster 1 and 2. Contrarily, the pressure pattern of cluster 3 with focal points of pressure on the heel and lateral side and moderate pressure on the forefoot is more present in DiabGr (10 diabetics vs. 8 controls). The low relative plantar pressure on Lateral in cluster 4 for DiabGr considerably deviates from the pressure distributions of the clusters for ContrGr. Consistently, this cluster is reflected in cluster 4 for BothGr. None of the session datasets from the controls were grouped into this cluster of 8 diabetics with neuropathy.

This study’s approach to modeling pressure distribution among the foot regions has several distinct aspects. First, *k*-medoids was used instead of *k*-means. Next to ensuring higher robustness towards outliers [23] (a shortcoming for which *k*-means is notorious), *k*-medoids allows for a more intuitive cluster representation: a cluster’s medoid is the most representative foot in the cluster, while a cluster centroid (under *k*-means) is a derived vector of averages that may be very different from any patient’s pressure distribution inside the cluster.

As shown in Fig 3C, one cluster (cluster 4) consists only of patient sessions, accounting for ca. 22% of sessions of diabetics. However, all other clusters contain both diabetics and controls. Does this imply that diabetics apply pressure in a similar way as controls in more than 78% of the sessions? Fig 2 provides an indication that this is not necessarily the case. In particular, Fig 2 shows that the quality of clusters over the control group is lower than the quality of the clusters over the patients, implying that controls exhibit much more diversity in plantar pressure distribution than patients. Hence, when clustering patients and controls together, the idiosyncrasies of the controls lead to clusters of poor quality, from which conclusions on the similarity between patients and controls should not be drawn. This is also supported by Fig 4C, where we see that the clusters built over both groups together are very close to each other, even overlapping.

Hence, the main conclusion is that there is one group of patients (cluster 4) that applies plantar pressure in a way that is not found amongst healthy controls. To draw conclusions about the other clusters, it is necessary to consider a larger sample of controls, that would allow to suppress the idiosyncrasies of individual controls and build homogeneous clusters of controls. Then, it may be tested whether the likelihood of observing patients in such a cluster of controls is increasing.

How stable are the clustering results for different choices of *k*? Fig 4D–4F juxtaposes the partitionings for clustering with different values of *k* on both groups. Notably, the overlap between different clusters is rather higher than for the optimum clustering run depicted in Fig 4C. As shown in Fig 4D–4F, the grouping with *k* = 3 is too rough. Adding a fifth or sixth cluster results in smaller clusters, but higher overlap. In general, higher *k* values reflect individual pressure patterns more accurately. However, very small groups may not represent significant pressure patterns as a result of overfitting.

To our knowledge, the design of the study protocol has not been similarly performed before to test for pressure distribution differences. The simplicity of the study protocol makes it easy to reproduce, however the complexity of foot movement within everyday life may not be captured by the protocol. Thus, there is a limitation of the study to only grasp differences of pressure distribution that may be detected by the changes of posture.

Another limitation of the findings is the small sample size. This study’s approach attempted to compensate for this limitation by (a) keeping the number of clusters small, so that the clusters do not reflect the idiosyncrasies of single patients and by (b) using *k*-medoids, which are less influenced by outliers than *k*-means (here: patients that apply pressure in a very different way than all the others). Nonetheless, the findings should be verified in a larger cohort.

Studying the differences between female and male participants was also prevented by the small sample size; the gender-specific samples would have been too small for generalization. A verification of the significant differences between female and male participants reported in [14] would require a larger sample, where differences in the pressure distribution between left and right foot can also be tested.

The restrictive protocol of this study revealed that both diabetics with severe neuropathy and controls apply pressure very differently, although the task of the study is very simple. When patients apply pressure in a non-controlled setting, the variance among their pressure profiles will inevitably increase. Hence, a study allowing for more elaborate activities of the patients (running, biking, walking up and down a stair, and so on) is needed to complement the findings.

Finally, a long term follow-up would allow to determine which patients develop ulcerations. This would constitute a decisive end-point and evaluation whether certain clusters are associated with higher risk for diabetic foot syndrome development.

## Conclusion

This study has identified three shared and one unique plantar pressure distributions between diabetics with severe polyneuropathy and a group of healthy controls in a study with a simplified protocol. Utilizing knowledge about different plantar pressure distributions by leveraging an alternative “data-driven” research approach has the potential to improve early detection, prevention and treatment of diabetic foot syndromes. However, researchers have to tackle several challenges to obtain meaningful results and new insights for clinical practice. Future studies have to answer whether significant differences in plantar pressure distributions amongst diabetics prevail or whether the identified patterns reflect idiosyncrasies of the data. A transfer of the results to everyday activities constitutes another demanding challenge.

## Supporting Information

S1 Datasets Plantar Pressure Study Datasets. (ZIP)
